# Efficacy and Safety of Eldecalcitol for Osteoporosis: A Meta-Analysis of Randomized Controlled Trials

**DOI:** 10.3389/fendo.2022.854439

**Published:** 2022-04-19

**Authors:** Hongyan Liu, Guoqi Wang, Ting Wu, Yiming Mu, Weijun Gu

**Affiliations:** ^1^Department of Endocrinology, The First Medical Center of Chinese PLA General Hospital, Beijing, China; ^2^Department of Pediatrics, The First Medical Center of Chinese PLA General Hospital, Beijing, China

**Keywords:** eldecalcitol, osteoporosis, randomized controlled trials (RCT), meta-analysis, vitamin D analog

## Abstract

**Object:**

Eldecalcitol (ED-71) is a vitamin D analog for the treatment of osteoporosis. However, inconsistent results have been reported in this regard. Hence, this meta-analysis of randomized controlled trials (RCTs) aimed to assess the efficacy and safety of ED-71 for osteoporosis.

**Methods:**

The PubMed, Embase, and the Cochrane Library databases were systematically searched to identify potential trials from inception until April 2021. The investigated outcomes included bone mineral density and fractures at various sites, and potential adverse events. The pooled effect estimates were calculated using weighted mean difference (WMD) and relative risk (RR) with 95% confidence interval (CI) using the random-effects model.

**Results:**

Eight RCTs involving 2368 patients were selected for the final meta-analysis. The pooled results showed that ED-71 were associated with a higher level of femoral neck (FN) bone mineral density (BMD) (WMD: 0.92; 95% CI: 0.24–1.60; *P* = 0.008), while it had no significant effect on lumbar spine BMD (WMD: 1.09; 95% CI: –0.11 to 2.30; *P* = 0.076) and hip BMD (WMD: 1.12; 95% CI: –0.16 to 2.40; *P* = 0.088). Moreover, the use of ED-71 could protect against the risk of all osteoporotic fracture (RR: 0.70; 95% CI: 0.55–0.88; *P* = 0.003) and vertebral fracture (RR: 0.74; 95% CI: 0.55–0.98; *P* = 0.038), while it did not affect the risk of nonvertebral fracture (RR: 0.53; 95%CI: 0.23–1.23; *P* = 0.140). The subgroup analyses found that the effects of ED-71 were superior to those of alfacalcidol on both BMD and fracture results. Moreover, the use of ED-71 plus bisphosphonate was associated with a greater improvement in BMD at various sites compared with bisphosphonate alone. Finally, ED-71 was associated with an increased risk of increased urine calcium level (RR: 1.69; 95% CI: 1.33–2.15; *P* < 0.001).

**Conclusion:**

This study found that the use of ED-71 could improve BMD and fractures at various sites, especially compared with alfacalcidol or a combination with bisphosphonate for patients with osteoporosis.

**Systematic Review Registration:**

[http://www.crd.york.ac.uk/prospero], identifier [CRD42021270536].

## Introduction

Osteoporosis is a chronic, progressive condition characterized by decreased bone mass and damaged microstructure of the bone, thus increasing the risk of skeletal fractures and accounting for more concerns in nowadays aging society (1). It has been reported that about 30% of all postmenopausal women are diagnosed as osteoporosis. And approximately 40% of these patients will go through skeletal fractures (2). More than 8.9 million fractures occurred due to osteoporosis worldwide (3). Osteoporosis can be divided into primary osteoporosis and secondary osteoporosis (4). Primary osteoporosis is an age-related metabolic disease. Its incidence increases with age, and it presents with many atypical clinical symptoms (5). The prevalence of primary osteoporosis in elderly individuals (> 60.0 years) was 36% in China; the incidence in women was higher than that in men (6). A previous study found that osteoporosis could cause serious consequences in terms of osteoporotic fractures, and the most common site was the vertebral body (7).

Currently, bisphosphonates are widely used for osteoporosis to prevent the risk of skeletal fractures (8). However, the bioavailability of bisphosphonates is lower, with less than 1% absorption in the gut. Moreover, after administering bisphosphonates, the patients cannot lie down within 30 min to reduce the topical effect on the gastric mucosa and esophageal regurgitation (9). These factors caused lower compliance and balanced the treatment effects of bisphosphonates. Eldecalcitol (ED-71) was developed in the early 1980s, which has already been approved for osteoporosis in Japan. In 2020, ED-71 was approved for osteoporosis of postmenopausal women in China. ED-71 could reduce the levels of biochemical and histological parameters for bone resorption (10). Numerous studies reported the treatment effectiveness of ED-71 for osteoporosis; however, inconsistent results were obtained (11–18). Therefore, the current study was performed based on randomized controlled trials (RCTs) to determine the efficacy and safety of ED-71 for patients with osteoporosis.

## Methods

### Protocol and Registration

Our meta-analysis was performed according to the Preferred Reporting Items for Systematic reviews and meta-analyses (PRISMA) recommendations. A protocol for this meta-analysis has been registered on PROSPERO (http://www.crd.york.ac.uk/prospero) and the registration number is CRD42021270536.

### Data Sources, Search Strategy, and Selection Criteria

This study was performed and reported according to the Preferred Reporting Items for Systematic Reviews and Meta-Analysis (19). Studies designed as RCTs and reporting the efficacy and safety of ED-71 for patients with osteoporosis were selected for meta-analysis. The publication language was restricted to English, while no restriction was placed on the publication status. The potential studies were identified using the following steps: (1) The PubMed, Embase, and the Cochrane Central Register of Controlled Trials were systematically searched throughout April 2021 using the following search terms: (eldecalcitol OR ED-71) AND (randomized controlled trials). The website http://clinicaltrials.gov/(US NIH) was applied to search trials that were already completed but not yet published. Then, the reference lists of relevant reviews or original articles were manually searched for further new eligible trials.

The literature search and study selection process were independently performed by two reviewers, and conflicts between reviewers were settled by mutual discussion until a consensus was reached. The inclusion criteria were as follows: (1) patients: osteoporosis or low bone mineral density (BMD)/osteopenia. Dual energy X-ray absorptiometry (DXA) was used to measure BMD. Osteoporosis is defined as lumbar spine (L1-4) BMD T-score was below -2.5 SD. Osteopenia is defined as L1-4 BMD T-score between -1.0 and -2.5 SD; (2) intervention: ED-71, irrespective of being used as monotherapy or combined with bisphosphonate; (3) control: placebo, alfacalcidol, or bisphosphonate; (4) outcomes: BMD at the lumbar spine, femoral neck (FN), or hip, all osteoporotic fractures, vertebral fractures, nonvertebral fractures, and potential adverse events; and (5) study design: all included studies having RCT design.

### Data Collection and Quality Assessment

Two reviewers independently abstracted the data from included trials following a standardized protocol. The collected information included first authors’ name, publication year, country, sample size, mean age, male proportion, body mass index, intervention, control, co-intervention, follow-up duration, and reported outcomes. The methodological quality of included trials was evaluated using the Cochrane Collaboration risk-of-bias tool, which included seven domains; each domain was assigned as low risk, unclear risk, and high risk (20). The data abstraction and quality assessment were independently performed by two reviewers, and inconsistencies between reviewers were settled by an additional reviewer referring to the original article.

### Statistical Analysis

The BMD at various sites was defined as continuous data, while fractures at various sites and adverse events were assigned as categorical data. Then, the weighted mean difference (WMD) and relative risk (RR) with 95% confidence interval (CI) were calculated in each trial before data pooling. After this, the random-effects model was used to calculate the pooled results, which considered the underlying variations across included trials (21, 22). The heterogeneity across included trials for each outcome was assessed using *I*^2^ and Q statistic; significant heterogeneity was defined as *I*^2^ > 50.0% or *P* < 0.10 (23, 24). The robustness of pooled results was assessed using sensitivity analysis by sequentially removing a single trial (25). The subgroup analyses for BMD and fractures at various sites were performed according to the intervention and control, and the difference among subgroups was assessed using the interaction *P* test (26). The publication bias for BMD and fractures at various sites was evaluated using funnel plots and Egger and Begg tests (27, 28). All reported *P* values were two sided, and the inspection level was 0.05. The quality of included trials was assessed using Review Manager (version 5.3), while quantitative analyses were performed using software Stata (Version 10.0; StataCorp, TX, USA).

## Results

### Literature Search

**Figure 1:**
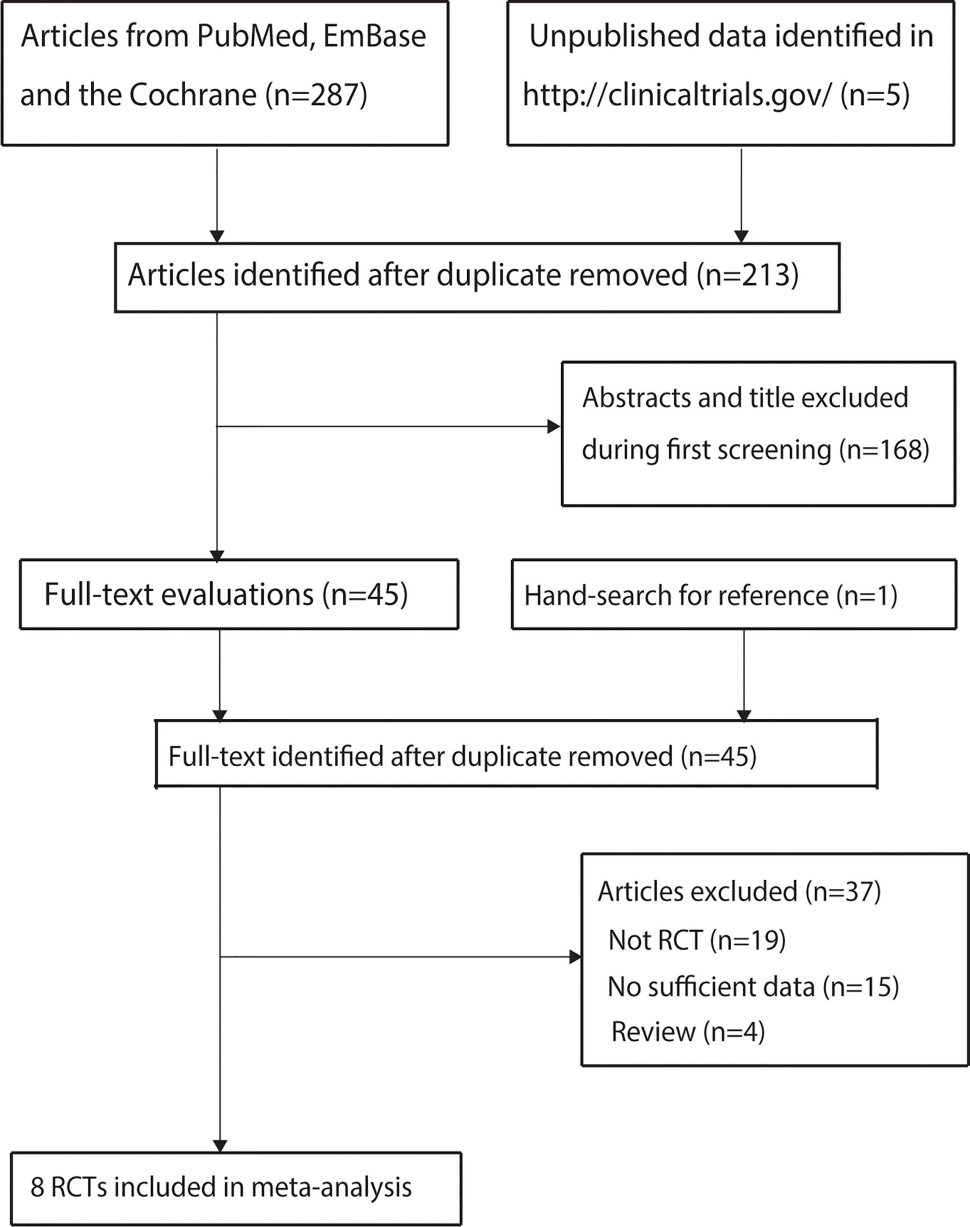
PRISMA Statement flowchart regarding the study selection process.

A total of 287 studies were identified from initial electronic searches, and 213 studies were retained after duplicate studies were removed. Further, 168 studies were excluded during title and abstract review because these studies reported irrelevant topics. The remaining 45 studies were retrieved for full-text evaluations, and 37 were removed because of the following reasons: not RCT (*n* = 19), no sufficient data (*n* = 15), and review (*n* = 4). The studies from other sources did not provide any new eligible trial. Finally, eight RCTs were selected for the final meta-analysis (9–16). The details of the study selection process are shown in **Figure 1**.

### Study Characteristics

The baseline characteristics of included studies are shown in **Table 1**. Seven trials were performed in Japan, and the remaining one trial was conducted in China. A total of 2368 patients were involved, and the sample size in an individual trial ranged from 28 to 1054. Three trials included only female patients, while the remaining five trials comprised both male and female patients. Three trials compared ED-71 with alfacalcidol, two trials compared ED-71 plus bisphosphonate with bisphosphonate alone, two trials compared ED-71 monotherapy with bisphosphonate, and the remaining one trial compared ED-71 with placebo. The follow-up duration for included trials ranged from 5.6 months to 36.0 months.

**Table 1:** Baseline characteristics of included studies.

Study	Country	Sample size	Age (year)	Male (%)	BMI (kg/m^2^)	Intervention and control	Co-intervention	Follow-up duration (month)
Matsumoto 2005 (11)	Japan	219	67.2	1.8	22.0	ED-71 0.5, 0.75, and 1.0 µg/day; placebo	Vitamin D_3_	12.0
Matsumoto 2011 (12)	Japan	1,054	72.2	2.3	22.2	ED-71 0.75 µg/day; ALF 1.0 µg/day	Vitamin D_3_	36.0
Sakai 2015 (13)	Japan	219	71.5	2.3	22.0	ED-71 0.75 µg/day plus alendronate 35 mg weekly; vitamin D 400 IU, calcium 610 mg daily plus alendronate 35 mg weekly	None	11.2
Nakatoh 2017 (14)	Japan	121	82.4	0.0	21.6	ED-71 0.75 µg/day; minodronate 50 mg/28 days; raloxifene 60 mg/day	None	11.2
Jiang 2019 (15)	China	249	65.5	2.8	22.6	ED-71 0.75 µg/day; ALF 1.0 µg/day	None	12.0
Suzuki 2019 (16)	Japan	28	67.3	0.0	20.3	Minodronate 50 mg/months plus ED-71 0.75 µg/day; minodronate 50 mg/month	None	18.0
Matsumoto 2020 (17)	Japan	360	58.4	33.6	NA	ED-71 0.75 µg/day; ALF 1.0 µg/day	None	24.0
Suzuki 2020 (18)	Japan	118	73.9	0.0	22.2	ED-71 0.75 µg/day; alendronate 35 mg weekly	None	5.6

ED-71, Eldecalcitol.

### Quality of Included Trials

The risk of bias for included studies are summarized in **Table 2**. All included trials were of moderate to high quality. Six trials reported a low risk of bias for random sequence generation, and allocation concealment. All trials reported a low risk of bias for incomplete outcome data, and selective reporting. Five trials reported a low risk of bias for blinding of outcome assessment, and three trials reported a low risk of bias for blinding of participants and personnel, and other biases.

**Table 2:** Risk of bias for included studies.

Study	Random sequence generation	Allocation concealment	Blinding of participants and personnel	Blinding of outcome assessment	Incomplete outcome data	Selective reporting	Other bias
Matsumoto 2005 (11)	Low risk	Low risk	Low risk	Low risk	Low risk	Low risk	Low risk
Matsumoto 2011 (12)	Low risk	Low risk	Low risk	Low risk	Low risk	Low risk	Unclear
Sakai 2015 (13)	Low risk	Unclear	High risk	Low risk	Low risk	Low risk	Unclear
Nakatoh (2017) (14)	Unclear	Low risk	High risk	High risk	Low risk	Low risk	Unclear
Jiang 2019 (15)	Low risk	Low risk	Low risk	Low risk	Low risk	Low risk	Unclear
Suzuki 2019 (16)	Low risk	Low risk	Unclear	Unclear	Low risk	Low risk	Low risk
Matsumoto 2020 (17)	Low risk	Unclear	High risk	Unclear	Low risk	Low risk	Low risk
Suzuki 2020 (18)	Unclear	Low risk	High risk	Low risk	Low risk	Low risk	Unclear

### Bone Mineral Density

**Figure 2:**
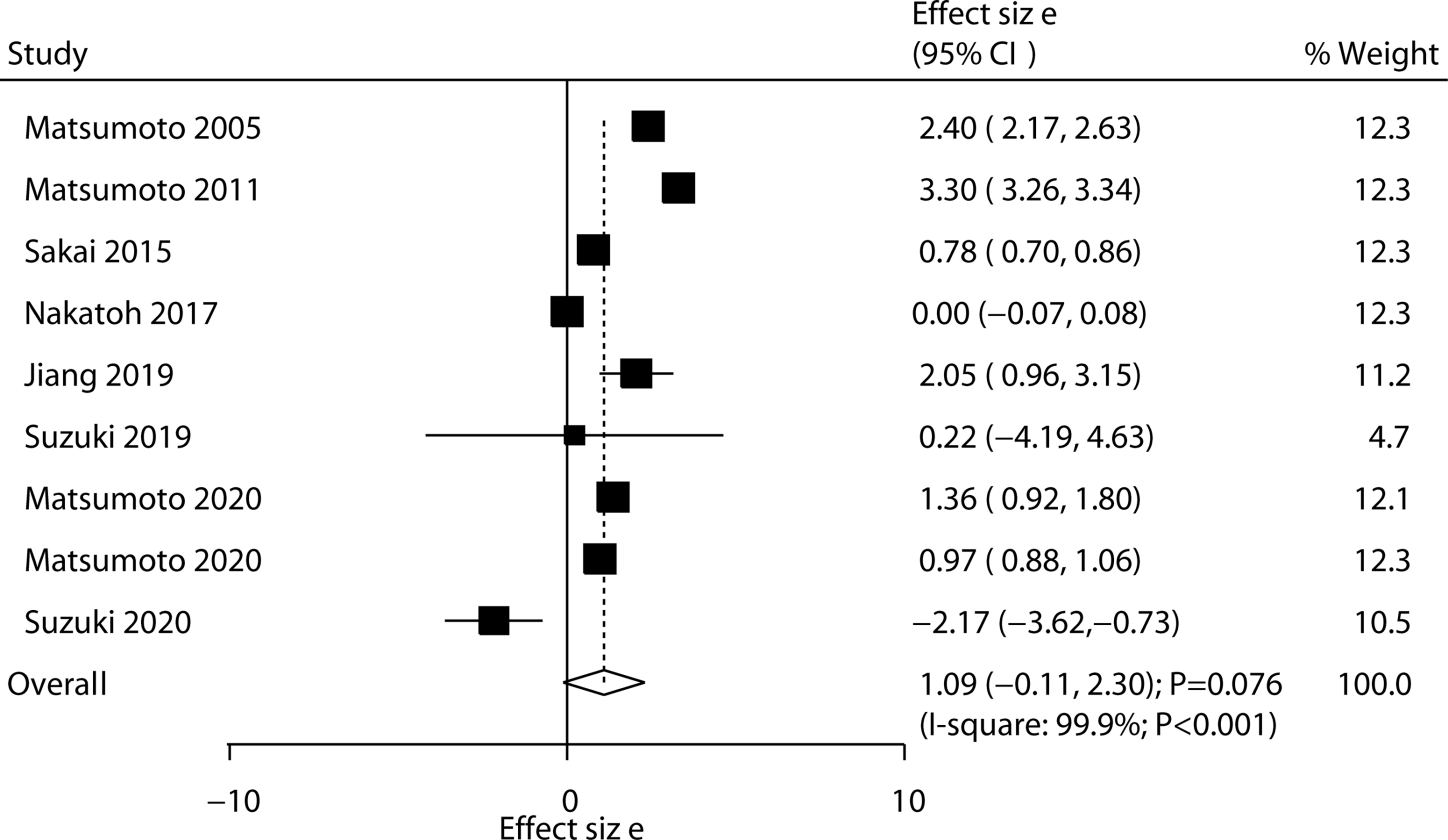
Effect of ED-71 on the change in lumbar spine BMD.

All included trials reported the effect of ED-71 on the change in lumbar spine BMD. After pooling all trials, we noted that ED-71 was not associated with the change in lumbar spine BMD (WMD: 1.09; 95% CI: –0.11 to 2.30; *P* = 0.076; **Figure 2**), and significant heterogeneity was observed across included trials (*I*^2^ = 99.9%; *P* < 0.001). The sensitivity analysis indicated that the pooled conclusion was variable because of marginal 95% CI (**Supplementary 1**). The subgroup analysis found that ED-71 was associated with high lumbar spine BMD compared with ED-71 versus placebo, ED-71 versus alfacalcidol, and ED-71 plus bisphosphonate versus bisphosphonate alone (**Table 3**). No significant publication bias for lumbar spine BMD was observed (*P* value for Egger: 0.379; *P* value for Begg: 0.602; **Supplementary 2**).

**Table 3:** Subgroup analyses for BMD and fractures at various sites.

Outcomes	Comparisons	WMD or RR and 95% CI	*P* value	Heterogeneity (%)/*P* value	Interaction *P* value
Lumbar spine BMD	ED-71 vs placebo	2.40 (2.17–2.63)	<0.001	–	<0.001
	ED-71 vs ALF	1.92 (0.30 –3.54)	0.020	99.9/< 0.001	
	ED-71 plus bisphosphonate vs bisphosphonate	0.78 (0.70–0.86)	<0.001	0.0/0.803	
	ED-71 vs bisphosphonate	−0.96 (–3.07 to 1.15)	0.373	88.4/0.003	
FN-BMD	ED-71 vs ALF	1.78 (0.18–3.38)	0.029	98.0/< 0.001	<0.001
	ED-71 plus bisphosphonate vs bisphosphonate	0.97 (–0.46 to 2.39)	0.185	73.1/0.054	
	ED-71 vs bisphosphonate	–0.41 (–1.44 to 0.62)	0.435	75.6/0.043	
Hip BMD	ED-71 vs placebo	0.00 (–0.19 to 0.19)	1.000	–	<0.001
	ED-71 vs ALF	1.81 (0.34–3.28)	0.016	99.9/< 0.001	
	ED-71 plus bisphosphonate vs bisphosphonate	0.14 (0.06–0.22)	0.001	0.0/0.927	
All osteoporotic fractures	ED-71 vs ALF	0.70 (0.54–0.89)	0.004	0.0/0.430	0.999
	ED-71 plus bisphosphonate vs bisphosphonate	0.69 (0.27–1.76)	0.438	–	
	ED-71 vs bisphosphonate	0.73 (0.08–6.72)	0.781	–	
Vertebral fracture	ED-71 vs ALF	0.71 (0.52–0.95)	0.021	0.0/0.952	0.252
	ED-71 plus bisphosphonate vs bisphosphonate	1.39 (0.45–4.27)	0.565	–	
Nonvertebral fracture	ED-71 vs ALF	0.65 (0.31–1.35)	0.248	36.0/0.210	0.139
	ED-71 plus bisphosphonate vs bisphosphonate	0.09 (0.01–1.14)	0.063	–	

**Figure 3:**
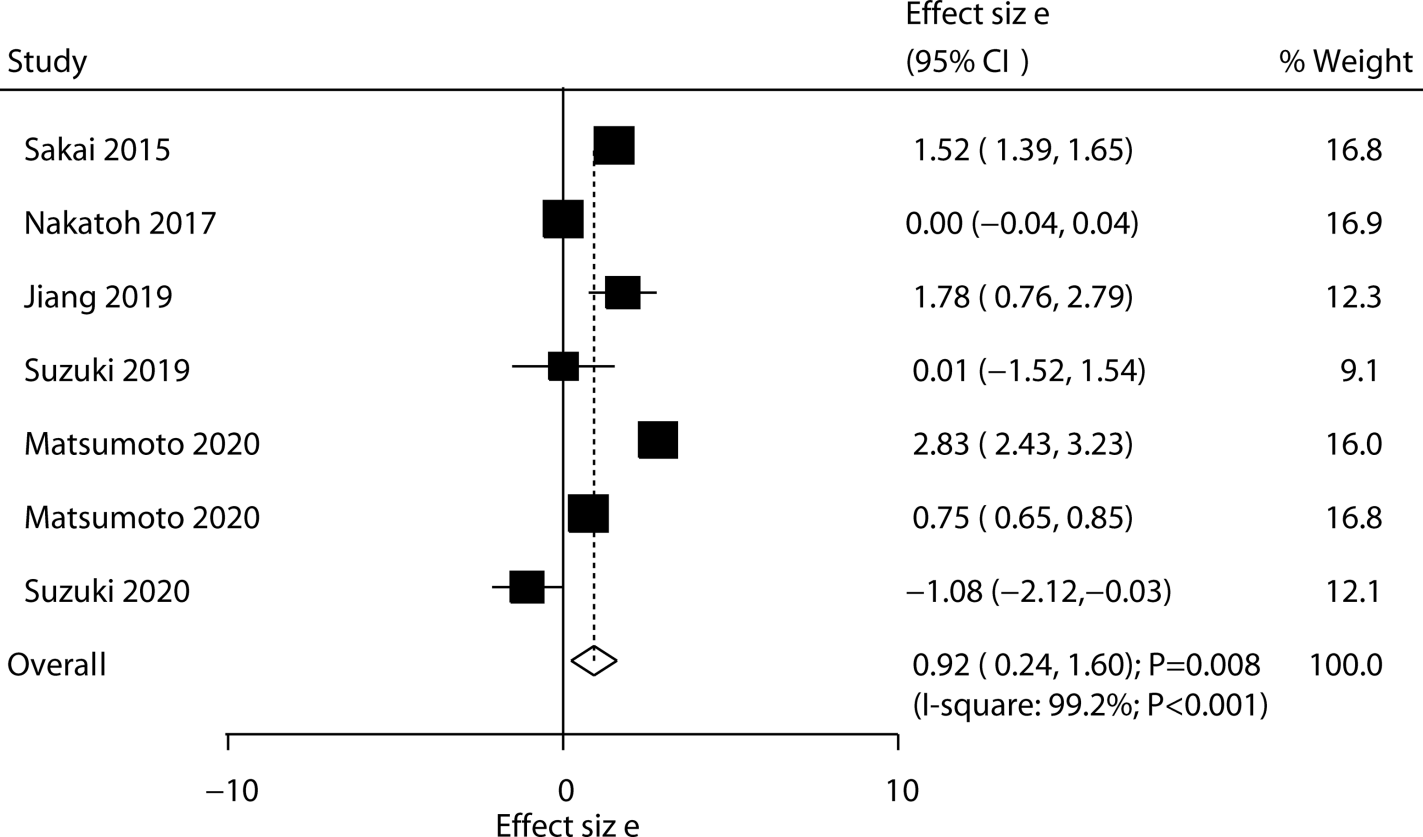
Effect of ED-71 on the change in FN-BMD.

Six trials reported the effect of ED-71 on the change in FN-BMD. The pooled result indicated that ED-71 was associated with a high level of FN-BMD (WMD: 0.92; 95% CI: 0.24–1.60; *P* = 0.008; **Figure 3**), and significant heterogeneity was detected among included trials (*I*^2^ = 99.2%; *P* < 0.001). The pooled conclusion was not stable because the lower limit was close to 0 (**Supplementary 1**). The subgroup analysis found that ED-71 significantly increased FN-BMD level compared with alfacalcidol (**Table 3**). No significant publication bias was found for FN-BMD (*P* value for Egger: 0.202; *P* value for Begg: 0.764; **Supplementary 2**).

**Figure 4:**
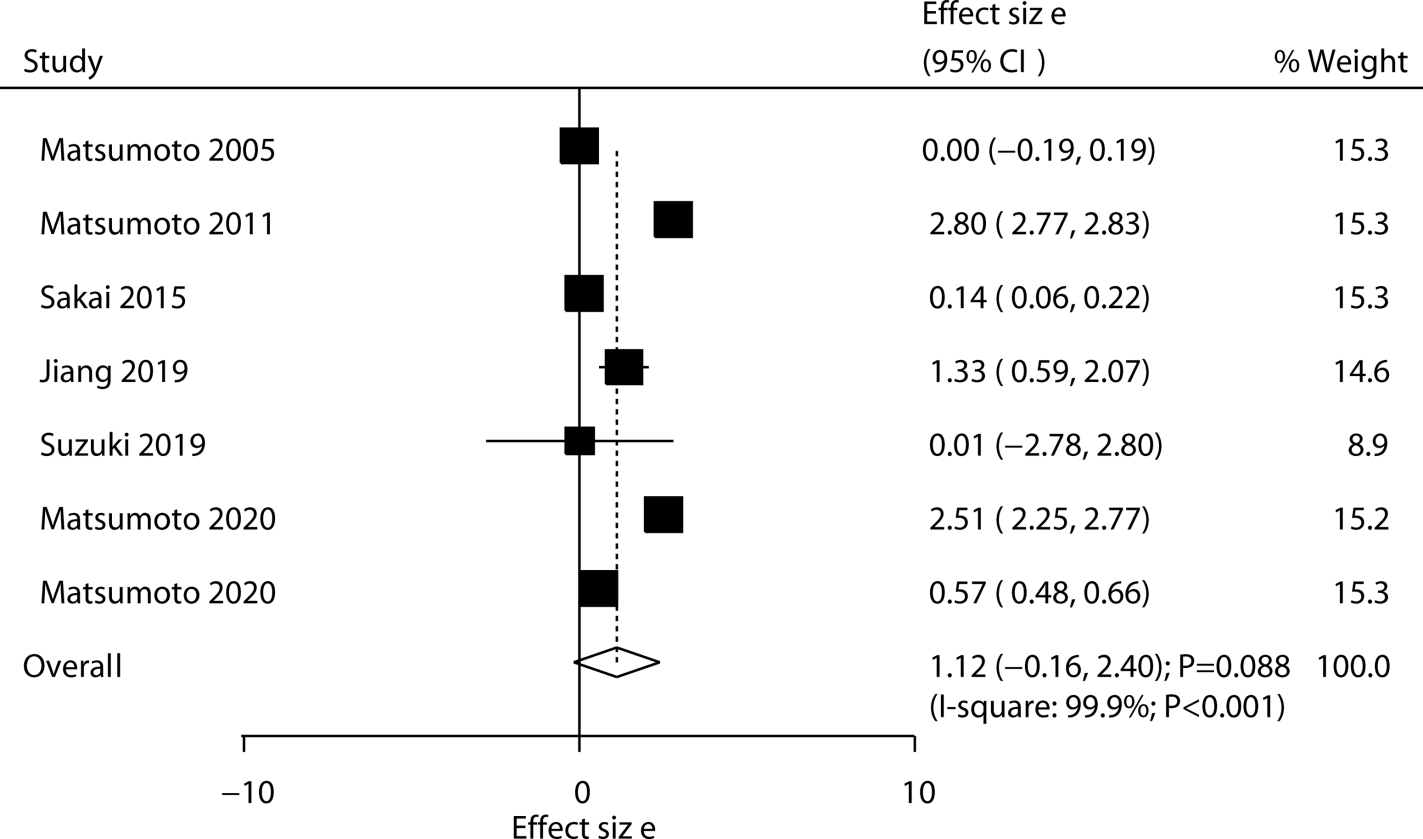
Effect of ED-71 on the change in hip BMD.

Six trials reported the effect of ED-71 on the change in hip BMD. No significant difference was observed between ED-71 and control for the change in hip BMD (WMD: 1.12; 95% CI: –0.16 to 2.40; *P* = 0.088; **Figure 4**), and significant heterogeneity was observed (*I*^2^ = 99.9%; *P* < 0.001). The sensitivity analysis suggested that ED-71 might exert a beneficial effect on hip BMD (**Supplementary 1**). The subgroup analysis found that ED-71 significantly increased hip BMD compared with a combination of ED-71 with alfacalcidol, and ED-71 plus bisphosphonate versus bisphosphonate alone (**Table 3**). No significant publication bias for hip BMD was observed (*P* value for Egger: 0.222; *P* value for Begg: 0.548; **Supplementary 2**).

### Fractures

**Figure 5:**
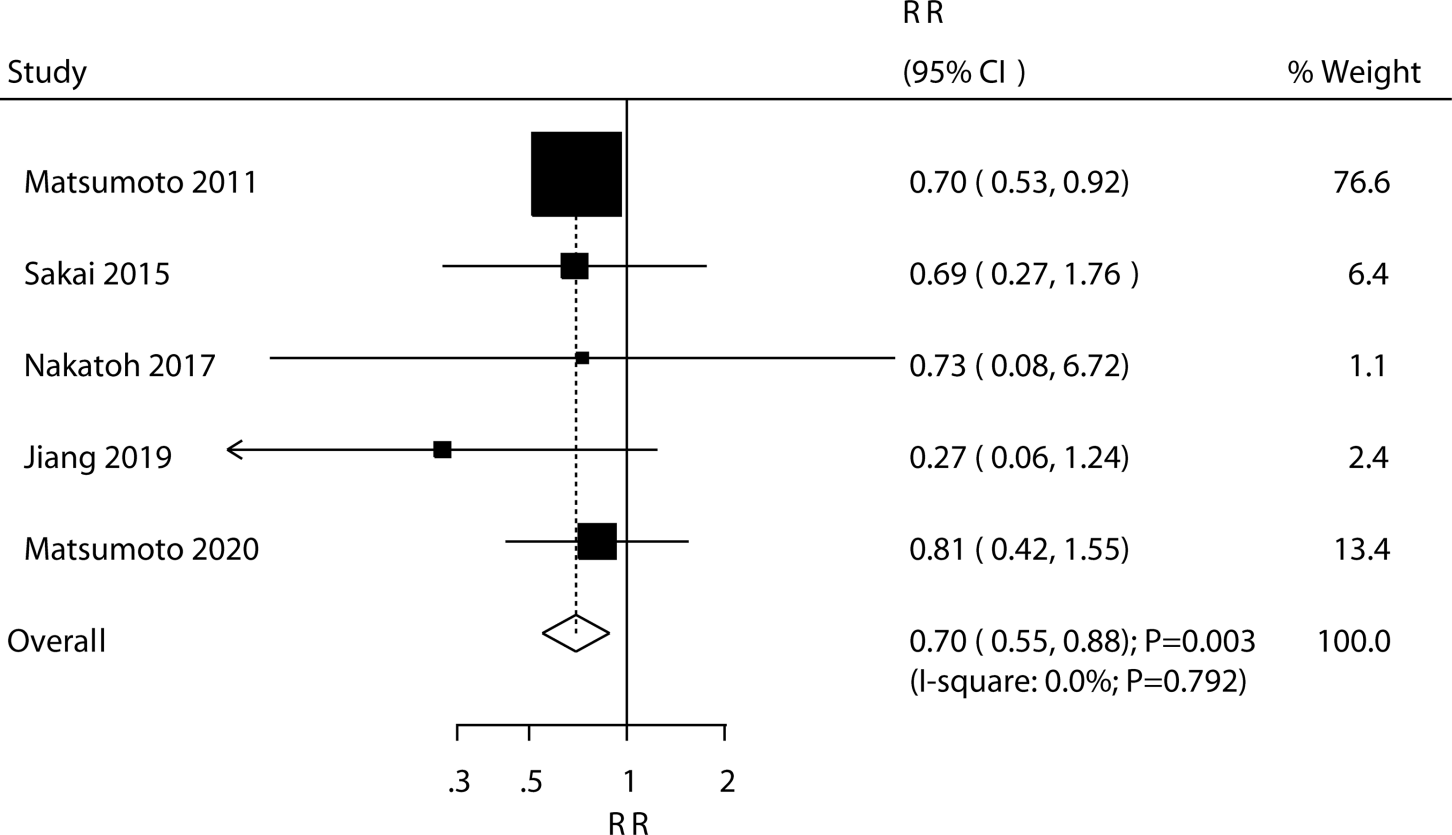
Effect of ED-71 on the risk of osteoporotic fractures.

Five trials reported the effect of ED-71 on the risk of all osteoporotic fractures; the use of ED-71 was associated with a reduced risk of all osteoporotic fractures (RR: 0.70; 95% CI: 0.55–0.88; *P* = 0.003; **Figure 5**). No evidence of heterogeneity was observed for all osteoporotic fractures (*I*^2^ = 0.0%; *P* = 0.792). The beneficial role of ED-71 in the risk of all osteoporotic fractures was not observed when removing the trial conducted by Matsumoto 2011, which specifically reported greater weight from the overall analysis (**Supplementary 1**). The subgroup analysis found that ED-71 significantly reduced the risk of osteoporotic fractures compared with alfacalcidol (**Table 3**). No significant publication bias was observed for all osteoporotic fractures (*P* value for Egger: 0.505; *P* value for Begg: 0.462; **Supplementary 2**).

**Figure 6:**
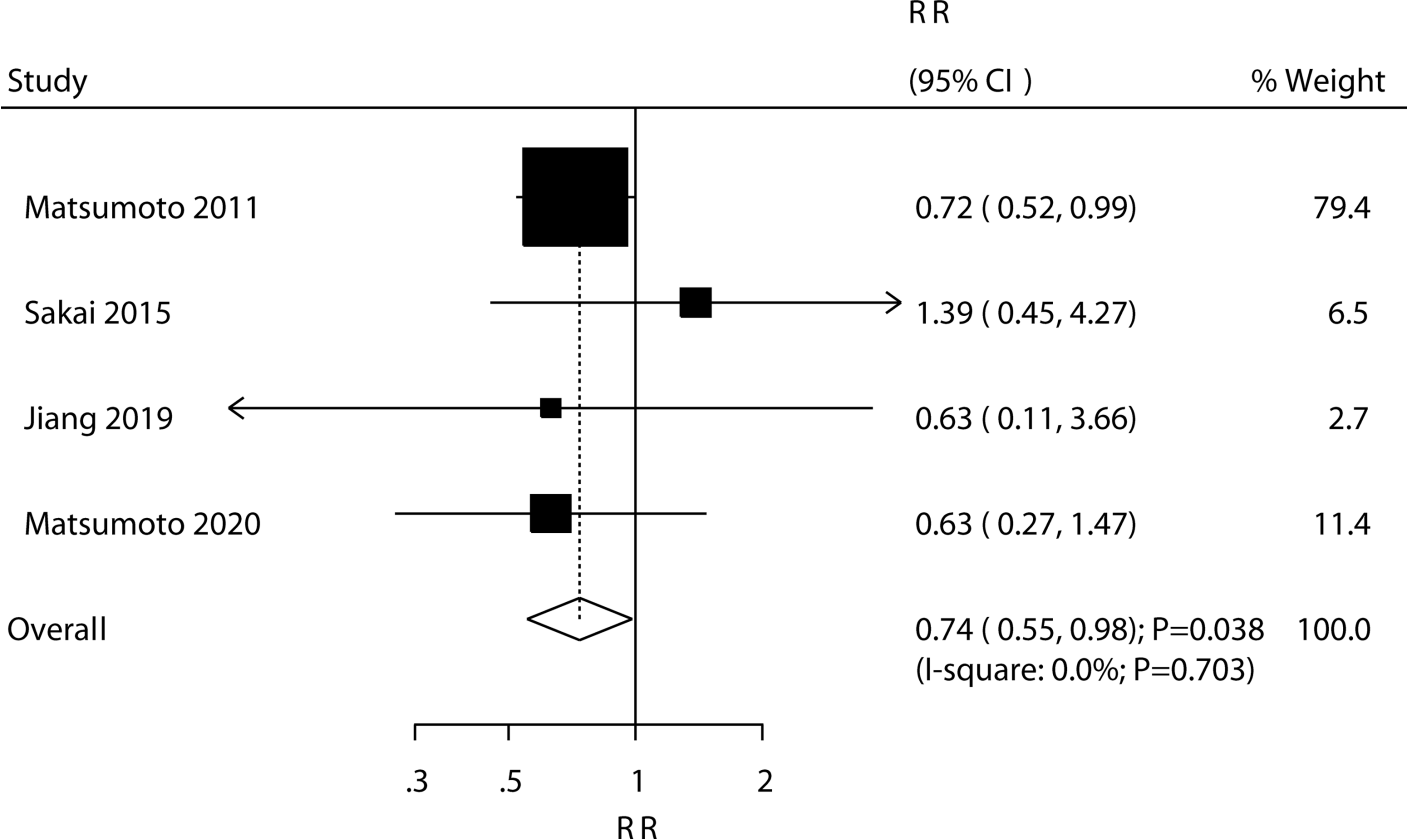
Effect of ED-71 on the risk of vertebral fractures.

Four trials reported the effect of ED-71 on the risk of vertebral fractures. ED-71 could protect against the risk of vertebral fractures (RR: 0.74; 95% CI: 0.55–0.98; *P* = 0.038; **Figure 6**), and no evidence of heterogeneity was observed (*I*^2^ = 0.0%; *P* = 0.703). The pooled result for vertebral fractures was not stable after sequentially removing a single study (**Supplementary 1**). The subgroup analysis suggested that ED-71 significantly reduced the risk of vertebral fractures compared with alfacalcidol (**Table 3**). No significant publication bias for vertebral fractures was observed (*P* value for Egger: 0.689; *P* value for Begg: 0.734; **Supplementary 2**).

**Figure 7:**
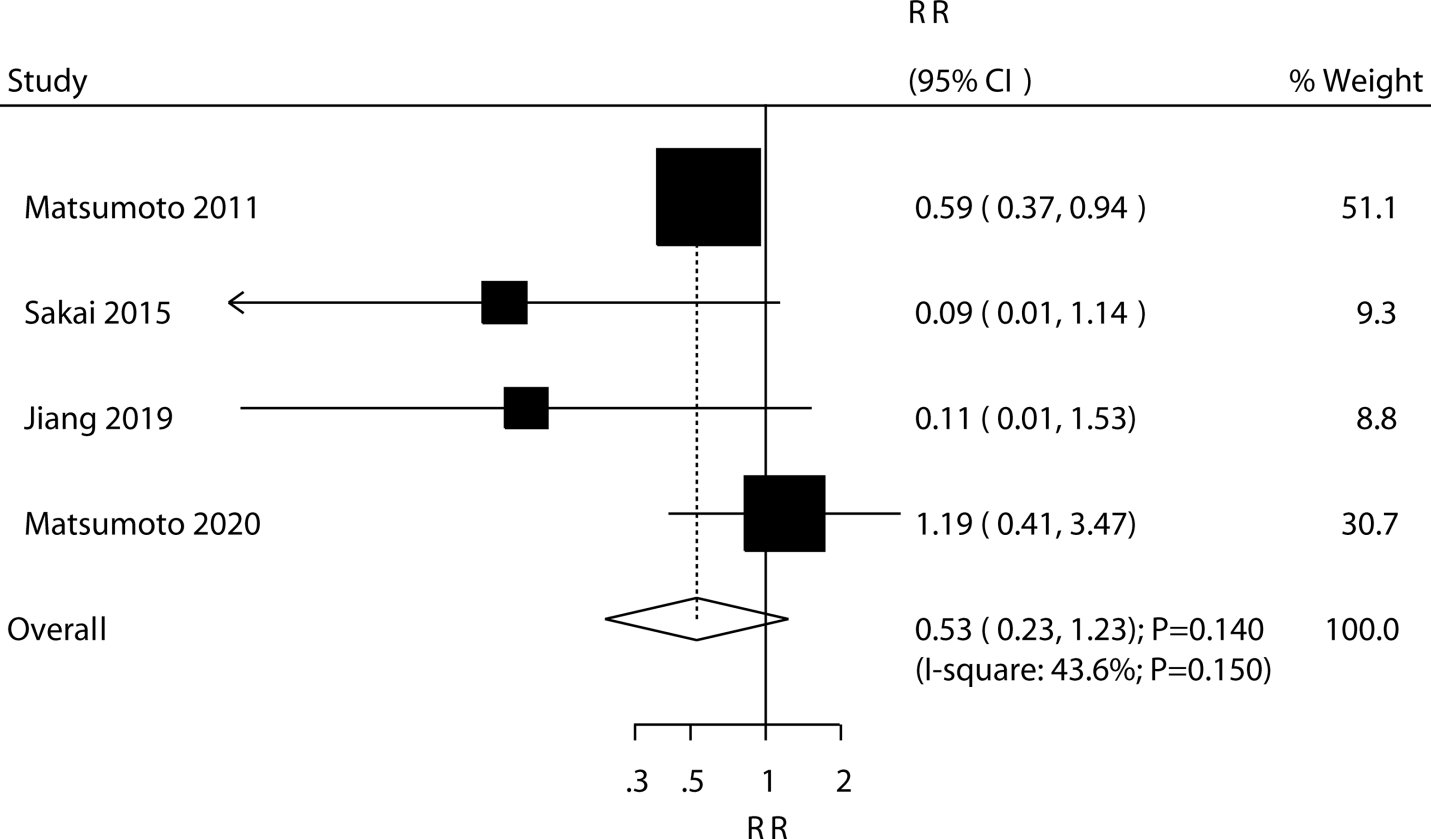
Effect of ED-71 on the risk of nonvertebral fractures.

Four trials reported the effect of ED-71 on the risk of nonvertebral fractures. The pooled result found that ED-71 was not associated with the risk of nonvertebral fractures (RR: 0.53; 95% CI: 0.23–1.23; *P* = 0.140; **Figure 7**). No significant heterogeneity for nonvertebral fractures was detected (*I*^2^ = 43.6%; *P* = 0.150). The sensitivity analysis indicated that the pooled conclusion was robust and not altered after excluding any particular trial (**Supplementary 1**). The subgroup analysis indicated that ED-71 was not associated with the risk of nonvertebral fractures, irrespective of the comparisons of ED-71 versus alfacalcidol, or ED-71 plus bisphosphonate versus bisphosphonate alone (**Table 3**).

### Adverse Events

The pooled results for adverse events are summarized in **Table 4**. ED-71 significantly increased the risk of the increase in urine calcium level (RR: 1.69; 95% CI: 1.33–2.15; *P* < 0.001). However, significant differences were found between ED-71 and control for the risk of discontinuation due to adverse events, headache, cystitis, increase in blood calcium level, nasopharyngitis, contusion, back pain, arthralgia, eczema, diarrhea, gastritis, pain in extremity, and dizziness.

**Table 4:** Pooled results for adverse events.

Outcomes	No. of trials	RR and 95% CI	*P* value	Heterogeneity (%)/*P* value
Discontinuation due to adverse events	3	0.76 (0.51–1.13)	0.175	0.0/0.372
Headache	2	1.34 (0.90–1.98)	0.151	0.0/0.365
Cystitis	2	1.20 (0.33–4.32)	0.778	48.9/0.162
Increase in blood calcium level	4	1.25 (0.68–2.32)	0.470	51.8/0.101
Increase in urine calcium level	4	1.69 (1.33–2.15)	< 0.001	0.0/0.407
Any serious events	5	0.91 (0.75–1.09)	0.285	0.0/0.587
Nasopharyngitis	3	1.05 (0.95–1.15)	0.323	0.0/0.496
Contusion	2	1.02 (0.82–1.28)	0.841	0.0/0.330
Back pain	2	0.91 (0.69–1.20)	0.504	0.0/0.599
Arthralgia	2	1.07 (0.77–1.48)	0.707	0.0/0.688
Eczema	2	0.43 (0.05–4.00)	0.461	78.8/0.030
Diarrhea	2	0.95 (0.40–2.25)	0.899	60.7/0.111
Gastritis	3	0.94 (0.28–3.10)	0.915	67.8/0.045
Pain in extremity	2	1.18 (0.41–3.40)	0.756	68.4/0.075
Dizziness	2	0.84 (0.55–1.30)	0.438	0.0/0.548

## Discussion

The present study was an updated meta-analysis to assess the efficacy and safety of ED-71 for osteoporosis based on RCTs comprehensively. A total of 2368 patients across a broad range of characteristics from eight RCTs were identified. This study found that ED-71 significantly increased FN-BMD and reduced the risk of all osteoporotic fractures and vertebral fractures. Moreover, the beneficial effects were mainly observed in the comparisons of ED-71 versus alfacalcidol, and ED-71 plus bisphosphonate versus bisphosphonate alone. Furthermore, the risk of the increase in urine calcium level significantly increased when using ED-71.

Several systematic reviews and meta-analyses already addressed the use of ED-71 for osteoporosis. Xu et al. performed a meta-analysis of three trials and found that ED-71 was associated with a high level of lumbar spine BMD and reduced the risk of vertebral fractures (29). However, the analysis was based on three RCTs, and several other efficacy outcomes were not addressed. Zheng et al. performed a meta-analysis of four studies and found that ED-71 plus bisphosphonate was associated with high FN-BMD and hip BMD after 6 months compared with bisphosphonate alone. Moreover, these effects were not observed after 12 months, while ED-71 plus bisphosphonate was associated with high lumbar spine BMD after 12 months compared with bisphosphonate alone (30). However, the analysis of this study was based on two RCTs and two observational studies, and the pooled results might be affected by uncontrolled biases. Therefore, we performed a meta-analysis of RCTs to evaluate the treatment effectiveness of ED-71 for osteoporosis.

The summary results indicated that ED-71 was superior to alfacalcidol for improving lumbar spine BMD, FN-BMD, or hip BMD, and reduced the risk of all osteoporotic fractures and vertebral fractures. The reason for these could be that ED-71 could reduce the number of preosteoblastic cells, which resulted in a reduction in the number and activity of osteoclasts on the bone surface through interacting with osteoclast precursors (31). Moreover, the “improvement in hip geometry and/or biomechanics in ED-71 was superior to that in alfacalcidol through increasing the cross-sectional area, volumetric BMD, and cortical thickness by mitigating endocortical bone resorption (32). However, ED-71 had no significant effect on the risk of nonvertebral fractures. This was because the number of events was lower than expected, and the power was not enough to detect a potential difference between ED-71 and alfacalcidol.

Compared with bisphosphonate alone, ED-71 plus bisphosphonate was associated with a greater increase in lumbar spine BMD and hip BMD. The use of bisphosphonate could improve the process of osteoporosis, while BMD continued to decline in nearly 15% of patients using bisphosphonate (33). Moreover, bisphosphonate could slow down the speed of bone gain with time (34). ED-71 could reduce the re-absorption of blood calcium in the intestine and improve the BMD for patients with osteoporosis (15). The use of ED-71 could decrease the bone resorption level for patients treated with bisphosphonate (35). Furthermore, superior effects of ED-71 plus bisphosphonate on the risk of fractures at various sites were not detected. This could be explained by the smaller number of trials included; also, the number of events was lower than expected. Finally, no significant differences were found between ED-71 and control for mostly reported adverse events. However, ED-71 was associated with an increased risk of the increase in urine calcium level, probably because it could reduce the re-absorption of blood calcium and the urine calcium level relatively increased.

Several shortcomings of this study should be acknowledged. (1) The heterogeneity for BMD at various sites were not fully explained by sensitivity and subgroup analyses. (2) The co-intervention of vitamin D and calcium were not consistent among included studies, which could affect the change in BMD and the risk of fractures. (3) The cause and severity of osteoporosis were different, and the improvement in BMD and fracture risk was affected. (4) The analysis was based on pooled data from published articles, the detailed analysis was restricted, and publication bias was inevitable.

In conclusion, this study found that ED-71 was superior to alfacalcidol in improving BMD at various sites and reduced the risk of all osteoporotic fractures and vertebral fractures. Moreover, ED-71 plus bisphosphonate was associated with a greater improvement in lumbar spine BMD and hip BMD compared with bisphosphonate alone. Further large-scale RCTs should be performed to assess the long-term treatment effects of BMD on fractures for patients with specific characteristics.

## Data Availability Statement

The original contributions presented in the study are included in the article/**Supplementary Material**. Further inquiries can be directed to the corresponding author.

## Author Contributions

HL and WG designed the study and edited the manuscript. HL, GW, and TW analyzed the data, performed the statistical analysis, and interpreted the data. YM edited and provided critical revision to the article. WG had primary responsibility for the final content. All authors read and approved the final manuscript.

## Conflict of Interest

The authors declare that the research was conducted in the absence of any commercial or financial relationships that could be construed as a potential conflict of interest.

## Publisher’s Note

All claims expressed in this article are solely those of the authors and do not necessarily represent those of their affiliated organizations, or those of the publisher, the editors and the reviewers. Any product that may be evaluated in this article, or claim that may be made by its manufacturer, is not guaranteed or endorsed by the publisher.
