# Supplementary material for: Efficacy and Safety of Eldecalcitol for Osteoporosis: A Meta-Analysis of Randomized Controlled Trials
Source: Front Endocrinol (Lausanne). 2022 Apr 19;13:854439. doi: 10.3389/fendo.2022.854439 (PMC9063410; doi:10.3389/fendo.2022.854439)
Supplement: Supplementary file 1 [file DataSheet_1.pdf]

Supplemental 1 legends:

Figure S1. Sensitivity analysis for lumbar spine BMD.

Figure S2. Sensitivity analysis for FN-BMD.

Figure S3. Sensitivity analysis for hip BMD.

Figure S4. Sensitivity analysis for all osteoporotic fractures.

Figure S5. Sensitivity analysis for vertebral fractures.

Figure S6. Sensitivity analysis for nonvertebral fractures.

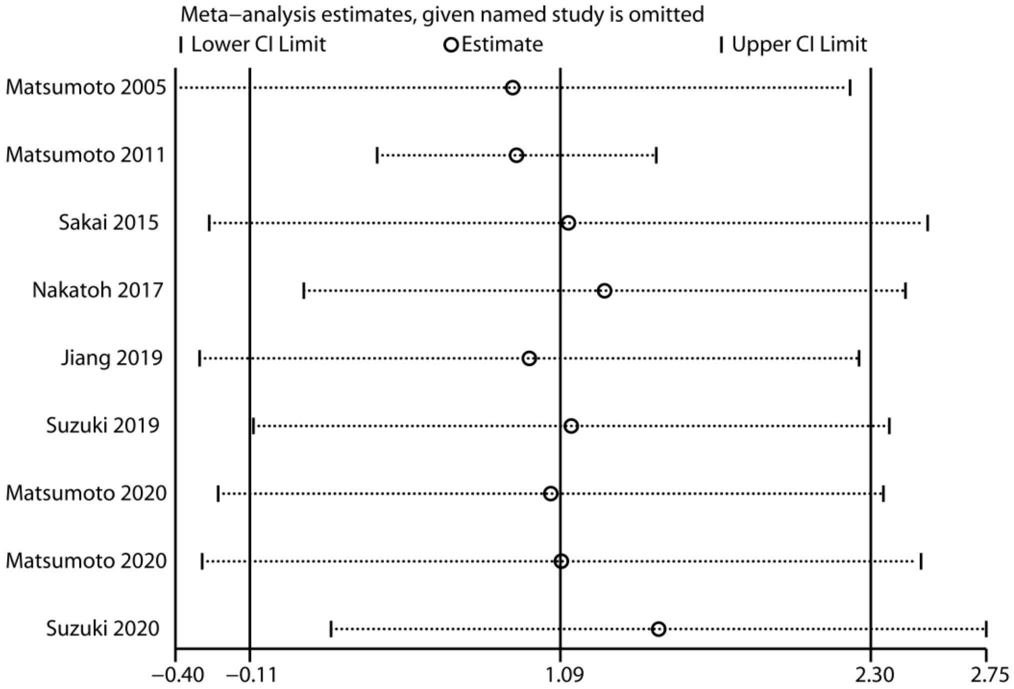

Figure S1. Sensitivity analysis for lumbar spine BMD.

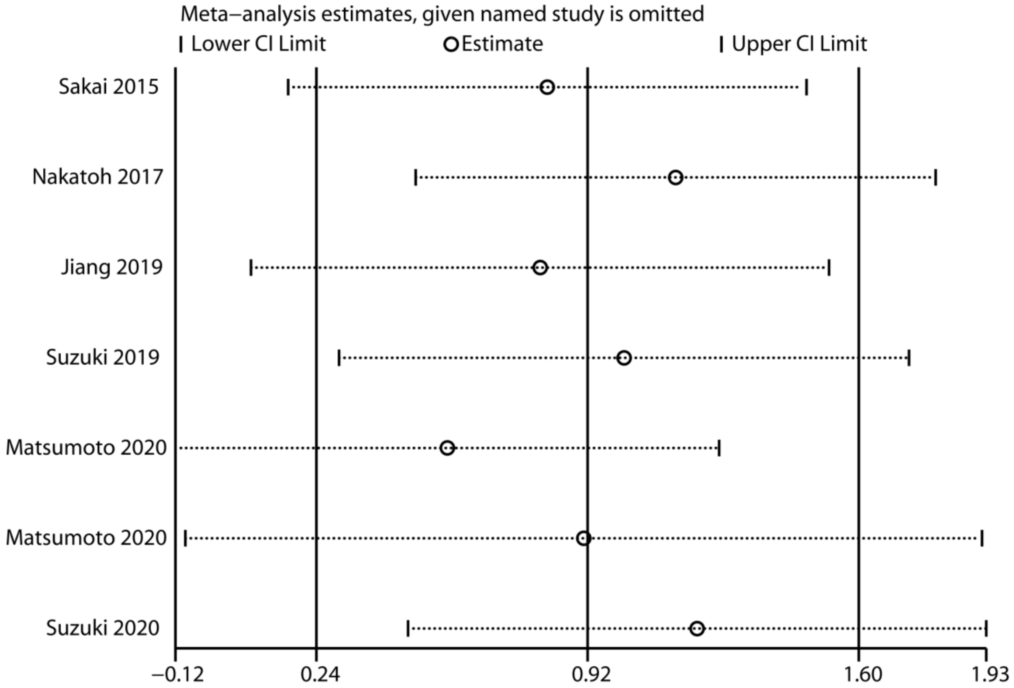

Figure S2. Sensitivity analysis for FN-BMD.

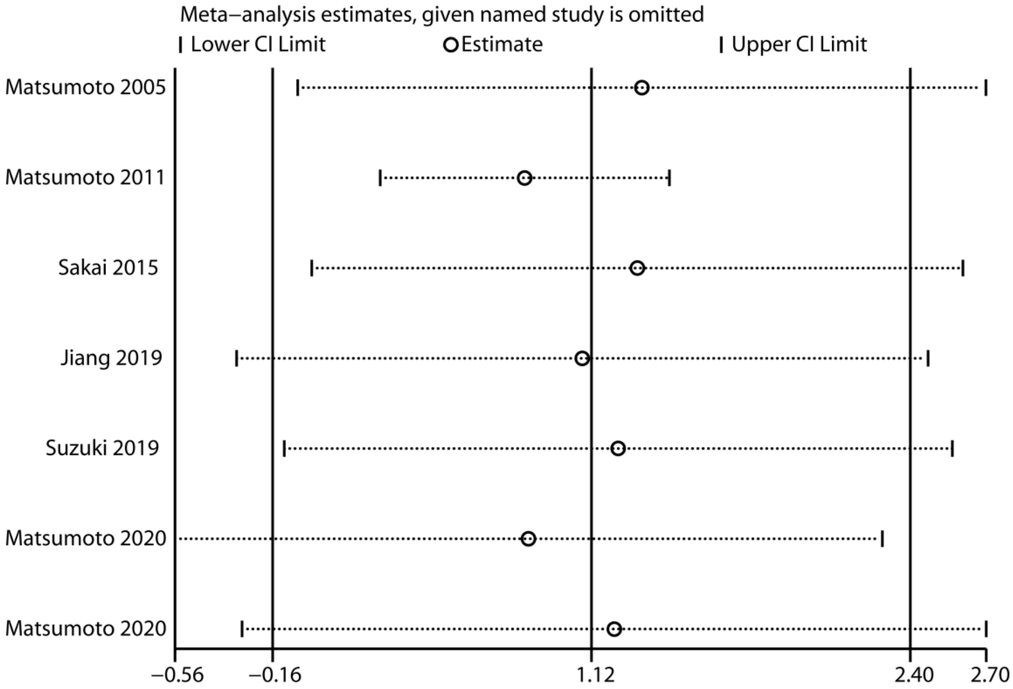

Figure S3. Sensitivity analysis for hip BMD.

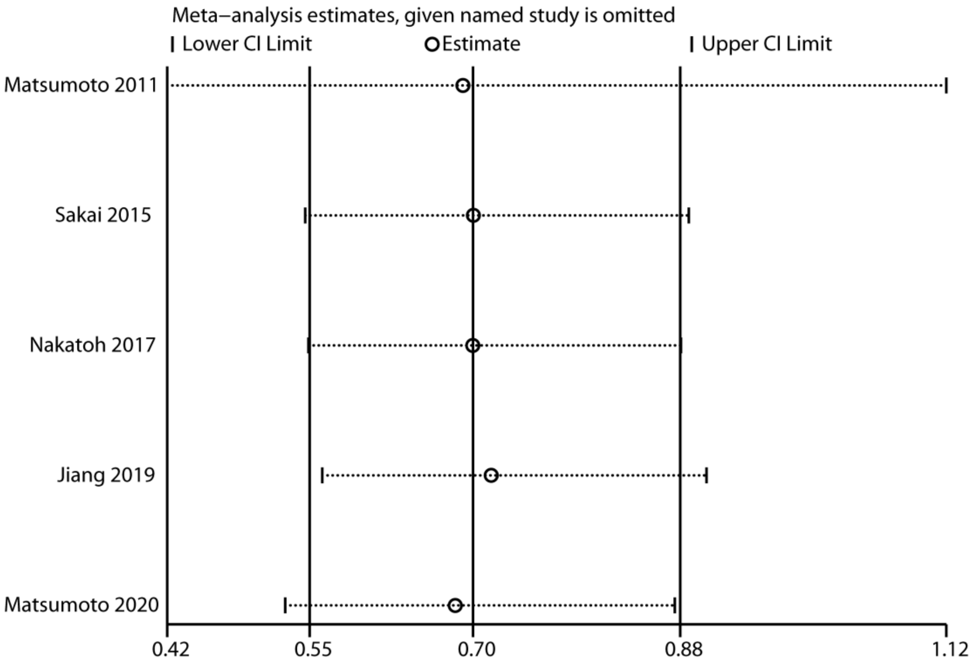

Figure S4. Sensitivity analysis for all osteoporotic fractures.

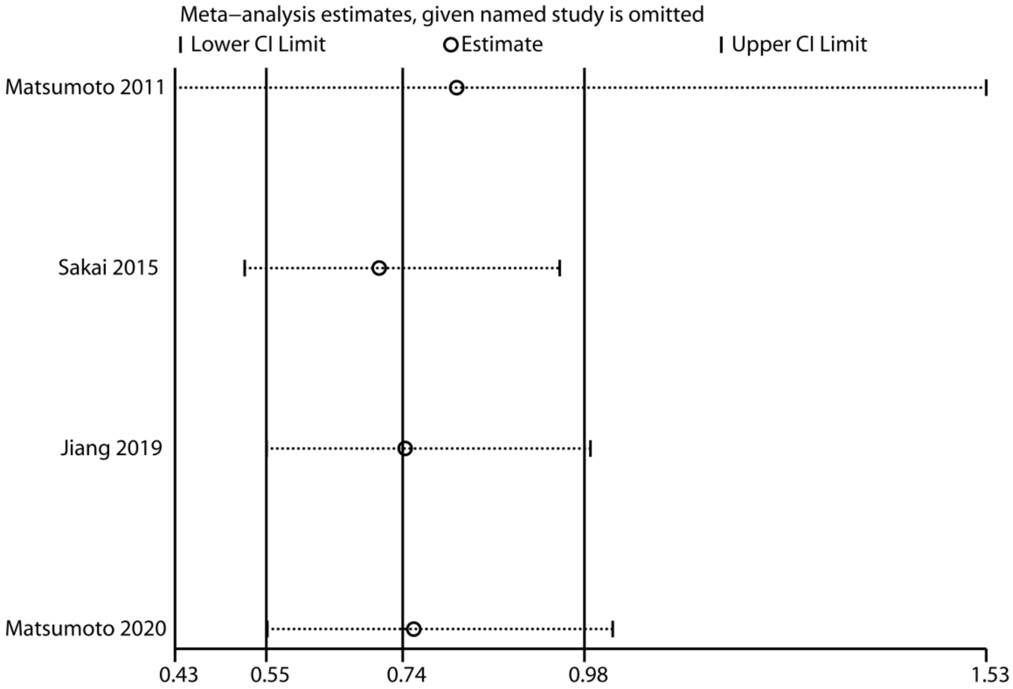

Figure S5. Sensitivity analysis for vertebral fractures.

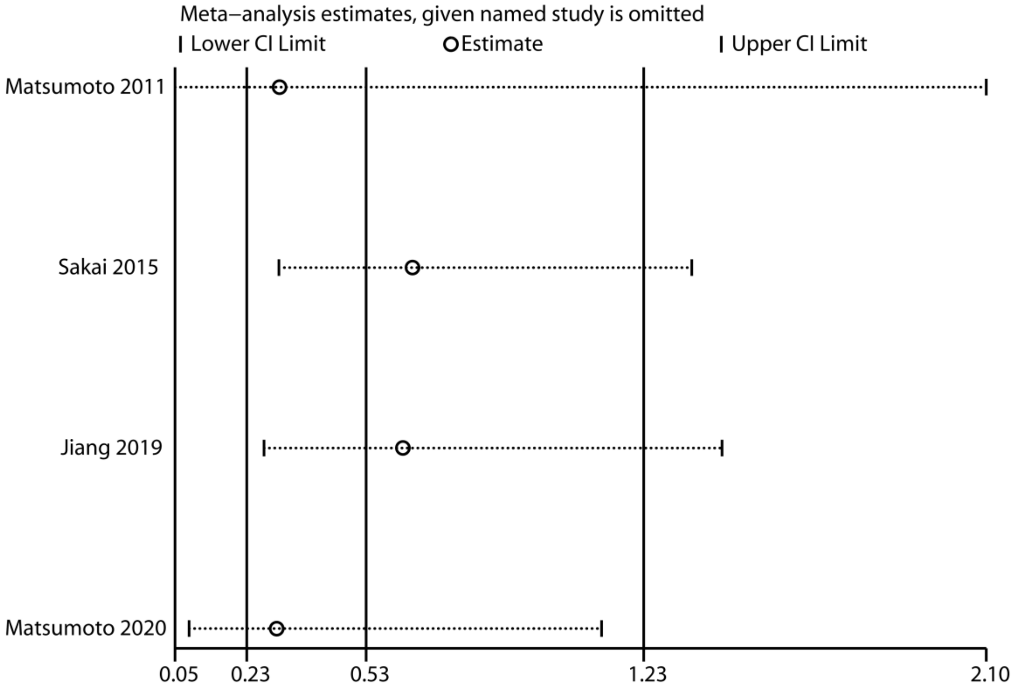

Figure S6. Sensitivity analysis for nonvertebral fractures.
