# Supplementary material for: Efficacy and Safety of Eldecalcitol for Osteoporosis: A Meta-Analysis of Randomized Controlled Trials
Source: Front Endocrinol (Lausanne). 2022 Apr 19;13:854439. doi: 10.3389/fendo.2022.854439 (PMC9063410; doi:10.3389/fendo.2022.854439)
Supplement: Supplementary file 2 [file DataSheet_2.pdf]

Supplemental 2 legends:

Figure S1. Funnel plot for lumbar spine BMD.

Figure S2. Funnel plot for FN-BMD.

Figure S3. Funnel plot for hip BMD.

Figure S4. Funnel plot for all osteoporotic fractures.

Figure S5. Funnel plot for vertebral fractures.

Figure S6. Funnel plot for nonvertebral fractures.

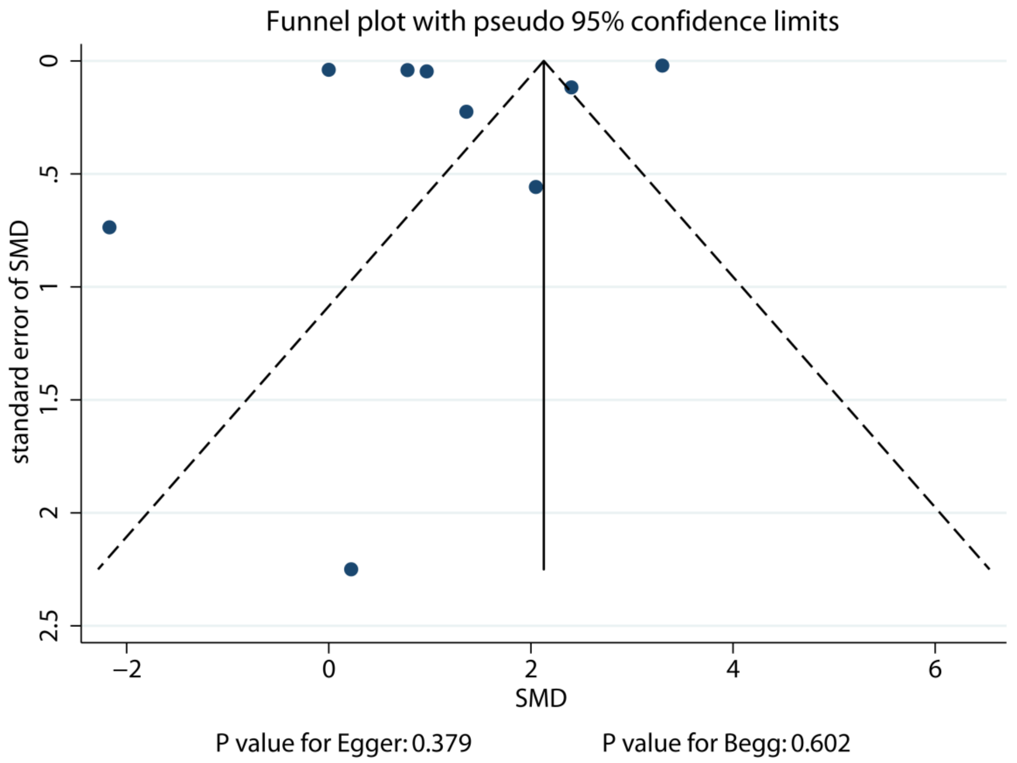

Figure S1. Funnel plot for lumbar spine BMD.

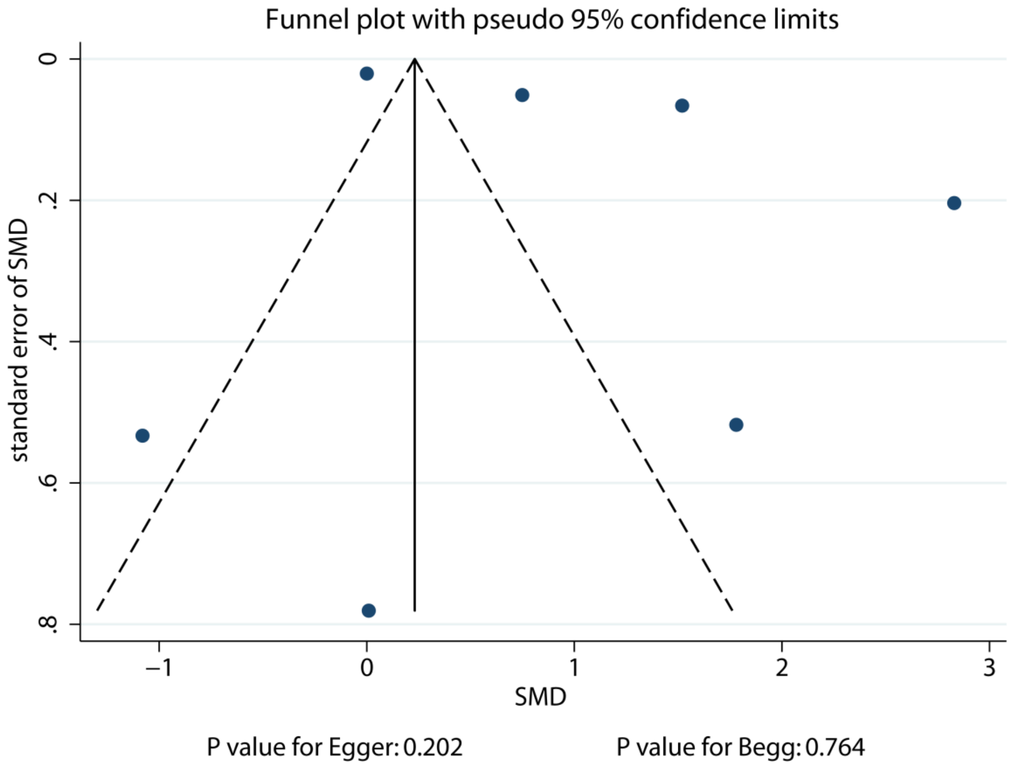

Figure S2. Funnel plot for FN-BMD.

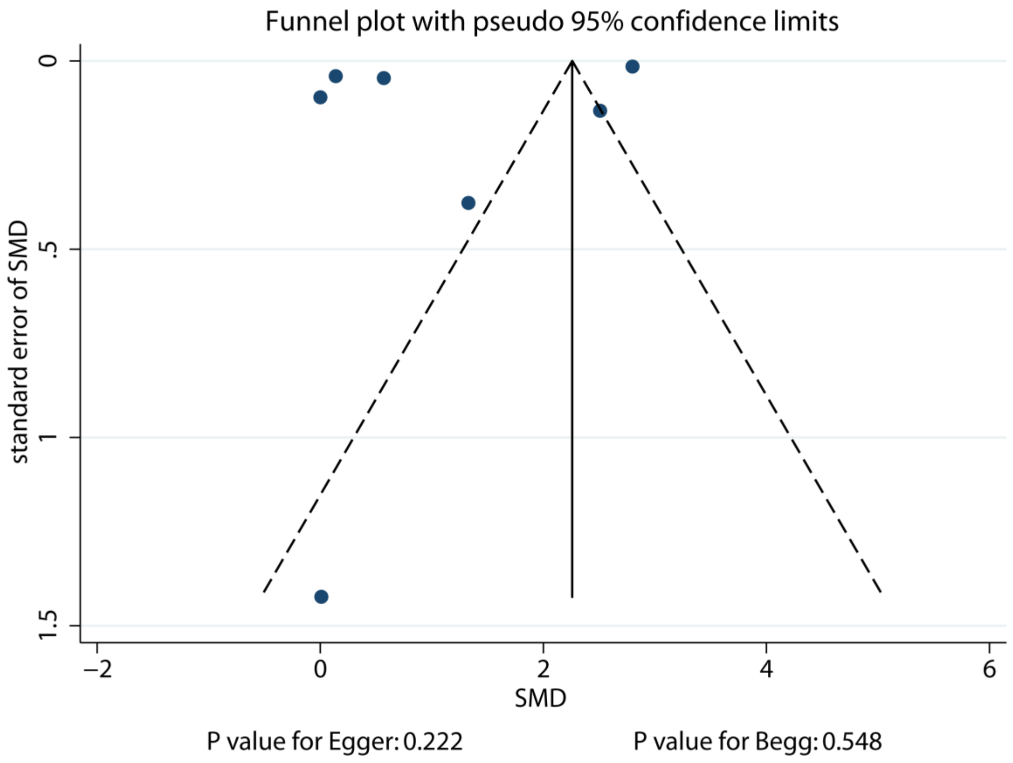

Figure S3. Funnel plot for hip BMD.

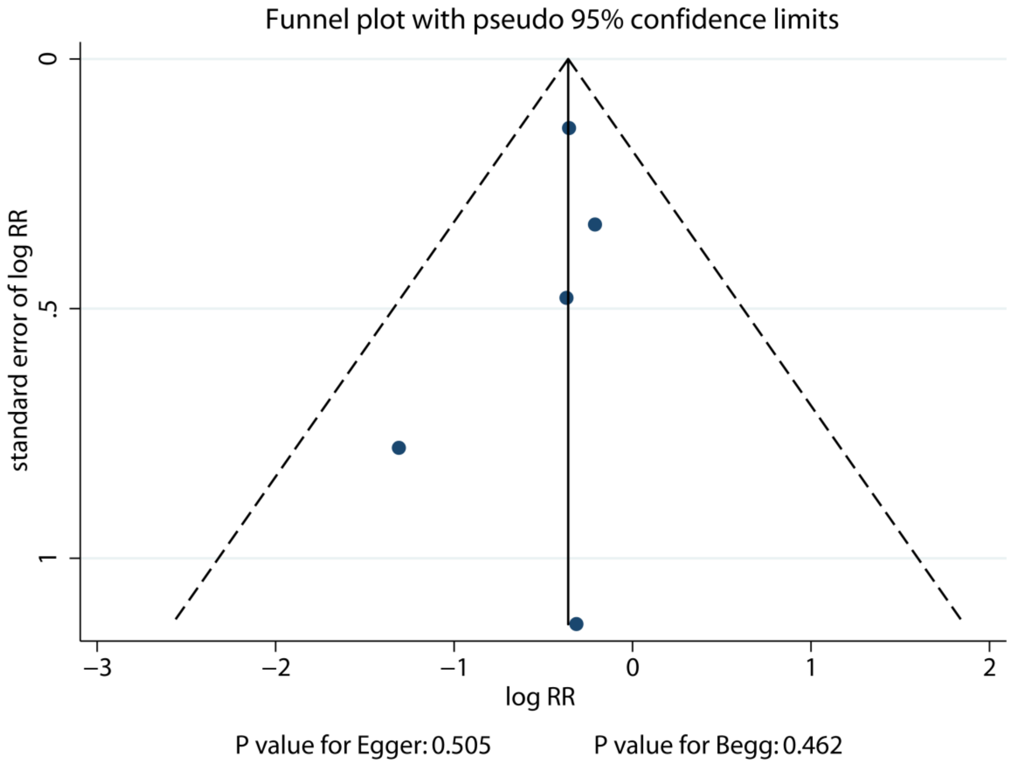

Figure S4. Funnel plot for all osteoporotic fractures.

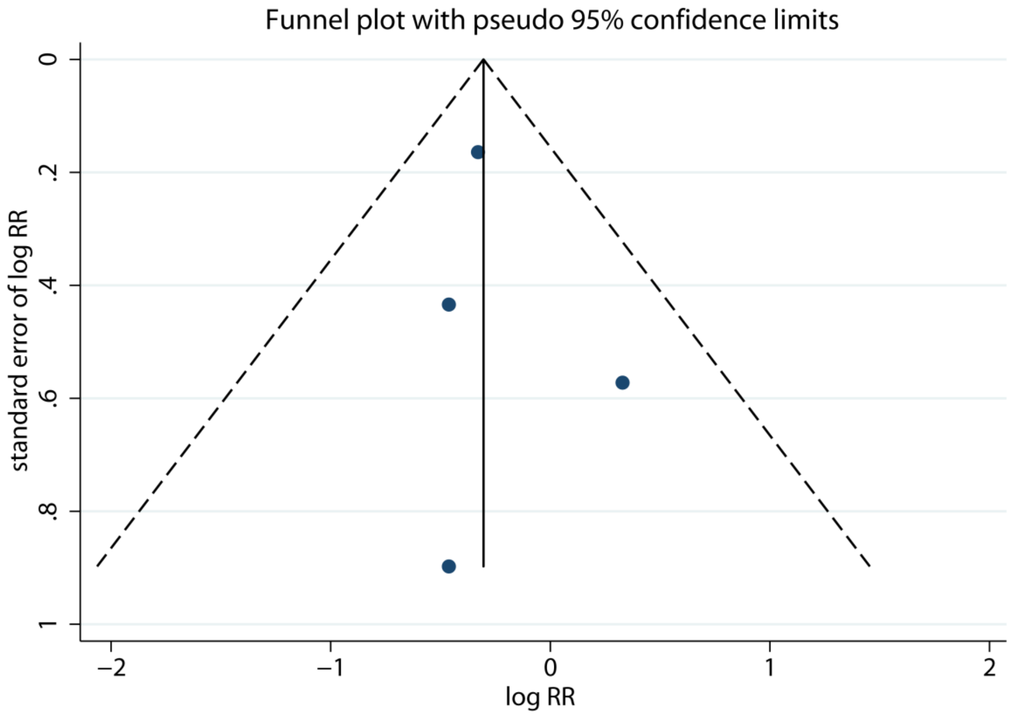

P value for Egger: 0.689 P value for Begg: 0.734

Figure S5. Funnel plot for vertebral fractures.

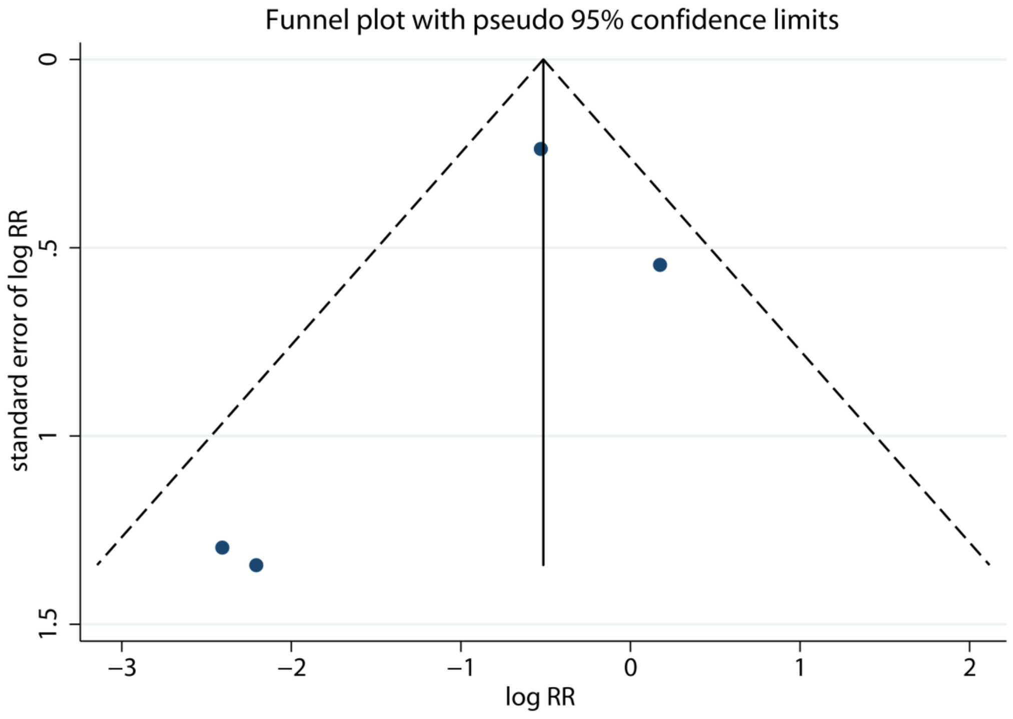

P value for Egger: 0.449 P value for Begg: 0.734

Figure S6. Funnel plot for non-vertebral fractures.
